# Morphological characteristics influence the spatial mixing patterns of shorebirds at Shengjin Lake

**DOI:** 10.1002/ece3.10054

**Published:** 2023-05-10

**Authors:** Chao Yu, Ruilin Zhang, Lizhi Zhou, Lei Cheng, Yiwei Bao, Yunwei Song

**Affiliations:** ^1^ School of Resources and Environmental Engineering Anhui University Hefei China; ^2^ Anhui Shengjin Lake Wetland Ecology National Long‐term Scientific Research Base Dongzhi China; ^3^ Anhui Province Key Laboratory of Wetland Ecosystem Protection and Restoration Anhui University Hefei China; ^4^ Department of Resources Conservation and Utilization Anhui Shengjin Lake National Nature Reserve Chizhou China

**Keywords:** functional feeding traits, mix‐species foraging, niche overlap, Shengjin Lake, wintering shorebirds

## Abstract

The coexistence of species with similar ecological niches is one of the core interests of community ecology research. However, how functional feeding traits, including bill size and leg length, determine the niche of mixed flocks of shorebird species has seldomly been studied, as well as, microhabitat variables affect the spatial patterns of availability and the quality of patches for wintering. From October 2016 to March 2017 at Shengjin Lake, Anhui Province, China, we recorded 226 scan samples from the different microhabitats and 93 focal animal videos of four common shorebird species: common greenshank, spotted redshank, Kentish plover, and little ringed plover. We found that the species participating in the mixed groups were different in each microhabitat. The results of the overlap index for microhabitats and foraging techniques between the species were consistent with the morphological characteristics of these species. Kentish and little ringed plovers had the highest Pianka's niche overlap index values of 0.95 and 0.98 for microhabitats and foraging techniques, respectively, whereas common greenshank and spotted redshank had values of 0.78 and 0.89, respectively. Common greenshank and spotted redshank used four foraging techniques: a single probe (PR), multiple probes (MPR), a single peck (PE), and multiple pecks (MPE). Kentish and little ringed plovers only used PE and MPE. The mean bill size, mean leg length, and mean foraging frequency were significantly associated with water depth. The mean bill size and mean leg length were both significantly correlated with the mean foraging frequency of shorebirds. The vegetated area was the most important variable for grouping among shorebirds. We concluded that the four species showed differences in their preferred microhabitats and foraging patterns. Interspecific morphological differences, including bill and leg lengths, resulted in niche differentiation. Thus, effective resource allocation by regional species was realized, and a dynamic balance was achieved by the mixed foraging species. The information on foraging behavior and habitat requirements could be useful in the management of water levels in natural areas and conservation of a diversity of wintering shorebirds.

## Graphical abstract

The four species (common greenshank, spotted redshank, Kentish plover, and little ringed plover) showed differences in their preferred microhabitats and foraging patterns. Interspecfic differences, including bill and leg lengths, resulted in niche differentiation.

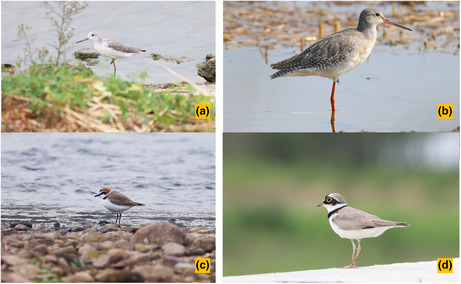

## 1 INTRODUCTION

The mechanism of coexistence of species with similar ecological niches, inhabiting the same space, has been a focus of community ecology studies since the theory of competitive exclusion was proposed (Barnagaud et al., 2021; De León et al., 2014). Niche separation induced by interspecific competition is an important factor that affects the coexistence of species in a community (Tarjuelo et al., 2017). Closely related species often avoid competition by dividing up important resources, making coexistence possible (Freeman et al., 2019). Niche overlap occurs when species use the same resources in similar ways (Ballejo et al., 2018; Colwell, 2010).

Bird species that coexist use resources, according to their ecological and morphological characteristics, from the different microhabitats within a habitat (Bai et al., 2021; Eckhardt, 1979) and thus have different habitat requirements (Zhang et al., 2014). Therefore, species with different ecological and morphological characteristics have developed separate ecological niches (Zhao et al., 2013), which decrease interspecific competition and ultimately lead to their coexistence (Xu et al., 2021). Ecological and morphological characteristics are related to specific behaviors between ecologically similar species and enable them to use different resources (Engelhardt et al., 2014; Schoener, 1974). Differences in bird bill shape (De León et al., 2014) or body size (Young et al., 2010) have been considered as an important limiting factor of the range of foraging techniques, microhabitat selection (Zhang et al., 2014), and dietary choices (Zhang et al., 2019). Long bills are associated with deep detection depth and for making sweeping motions in the water, whereas short bills are associated with pecking and probing on the surface of substrates (Pandiyan & Asokan, 2016). The functional requirement of tactile foraging strategies is high permeability, which is influenced by morphological characteristics. Shorebirds usually assemble in mixed‐species flocks and feed on various wetlands (Cheng et al., 2020). Foraging‐based ecological niche separation in shorebirds (Baker & Baker, 1973; Novcic, 2016) has aroused widespread interest.

The Yangtze River floodplain is unique in its extensive ephemeral river–lake wetlands, which are important wintering grounds for tens of thousands of shorebirds on the East Asian–Australasian flyway. Some wetlands, especially the shallow river–lake connected lakes, such as Shengjin Lake, are important stopovers and wintering sites. Shorebirds at Shengjin Lake are mostly distributed in the surrounding shallow water areas. However, the water level of Shengjin Lake is controlled by sluice gates, which results in a dramatic change of suitable foraging habitats (Fox et al., 2011; Zhang et al., 2021) and forces waterbirds to congregate in smaller areas (Yu et al., 2019).

We studied the mixed‐species patterns of migrating shorebirds from the perspective of foraging‐based ecological niches and described their foraging strategies. We aimed to verify whether: (a) ecological niche separation occurs on account of microhabitat selection; (b) the differences in foraging technique facilitate shorebird coexistence; and (c) foraging by mixed species is related to the differences in foraging behavior, brought about by morphological characteristics.

## 2 MATERIALS AND METHODS

### 2.1 Study area

Shengjin Lake (116°55–117°15E, 30°15–30°30N) is a river‐connected lake, with an area of 13,300 hectares. It is situated on the East Asian–Australasian flyway and is a wetland of international importance (https://rsis.ramsar.org/ris/2248). The rainy and dry seasons last from April to October and from November to March, respectively. Annual water levels fluctuate, increasing and dropping during the rainy and dry seasons, respectively. In the dry season, large mudflats in the lake serve as foraging habitats for wintering shorebirds. The lake is separated by sluice gates, and the water level is artificially controlled, which causes a particularly rapid rise and descent of water levels leading to flooding or a rapid exposure of the mud flats. The foraging and distribution of shorebirds is strongly influenced by these rapid changes which result in the formation of many habits of varying depth and size.

### 2.2 Microhabitat types

The water depth of the lake increases gradually from the shore to the water body, and shorebirds choose patches with different depths for foraging, according to the lengths of their legs and bills. The foraging microhabitats were classified into the following types: (H1) vegetated area, vegetation that grows on mud‐ or sandflats, with a height not exceeding the bird's body height; (H2) dry mud, which birds can walk across and not sink; (H3) soft mud, which is covered by surface water with a depth of 0–0.5 cm, where birds can sink; (H4) shallow water, with a water depth of 0.5–3 cm; (H5) intermediate water depth, with a water depth of 3–6 cm; (H6) deep water, with a water depth of 6–9 cm; and (H7) the water body, with a depth of >9 cm.

### 2.3 Foraging sampling

**FIGURE 1:**
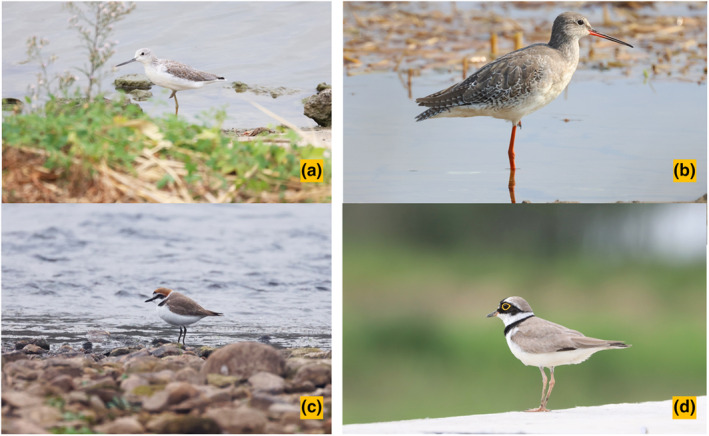
Four common shorebird species with different bill and leg length at Shengjin Lake. (a) Common greenshank, (b) spotted redshank, (c) Kentish plover, and (d) little ringed plover, respectively. Credit: Chao Yu.

**FIGURE 2:**
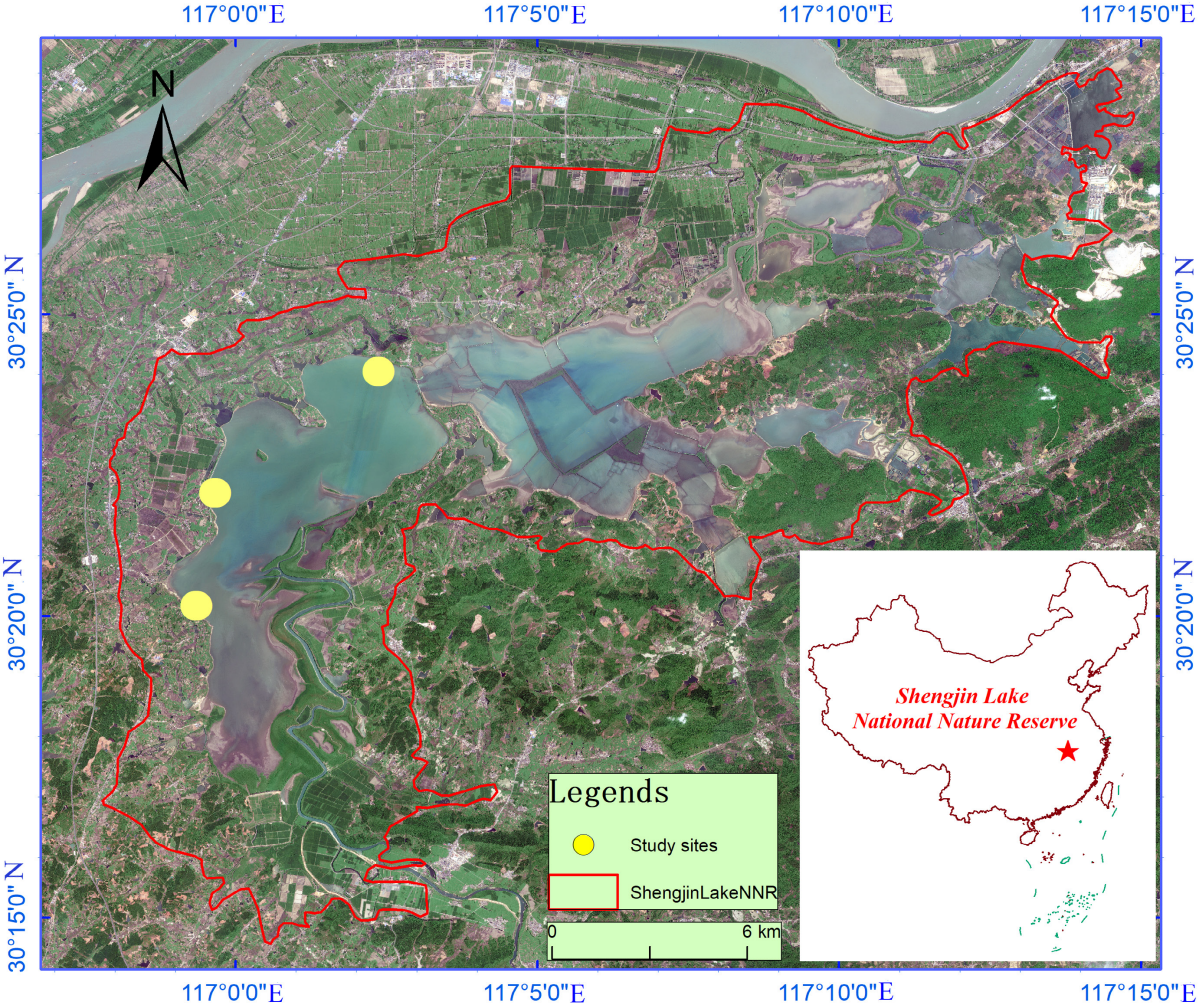
Location and study sites distribution map of Shengjin Lake National Nature Reserve.

A comprehensive survey of the four foraging shorebird species with different bill and leg length: common greenshank (*Tringa nebularia*), spotted redshank (*Tringa erythropus*), Kentish plover (*Charadrius alexandrinus*), and little ringed plover (*Charadrius dubius*) (Figure 1), was conducted on the mudflats of Shengjin Lake (Figure 2). These species forage in microhabitats that consisted of H1–H7. The fieldwork began in October 2016 and ended in March 2017. Foraging samples were collected daily from 7:00 to 17:00. We did not record observations during thick fog, strong winds, or heavy snow to reduce the influence of severe weather on observation results.

We rapidly recorded total number of individuals, foraging individual number, and individuals' instantaneous foraging techniques for each species in mixed‐flock shorebird groups by sequentially scanning habitats with a ZEISS video telescope (Victory PhotoScope 85 T*FL, 15–45×). Scanned videos that included clearly identifiable individuals and instantaneous foraging techniques were collected every 30 min in the three sites that consisted of seven microhabitat types (H1–H7). The instantaneous foraging techniques of birds were classified into the following types by using the PIPI Player (version 3.4.0, Ku6 Media Co., Ltd.) at a playback speed of 0.5× to observe them: a single peck (PE, a single movement of gathering food from the soil and water surface by pecking the substrate with less than one‐quarter of the length of their bill); multiple pecks (MPE, multiple repeated movements by pecking the substrate with less than one quarter of the length of the bill); a single probe (PR; a single movement of penetration into the soil or water surface with more than one quarter of the length of the bill); and multiple probes (MPR; multiple repeated movements of penetration into the soil or water surface with more than one quarter of the length of the bill) (Baker & Baker, 1973). We recorded 226 scan samples for interpretation from the different microhabitats (File S1).

Foraging individuals were randomly selected and their foraging behaviors (foraging time in 5 min; foraging frequency (number of pecks or probes in 5 min); a foraging bout length (total time for searching for food, handling, and swallowing food)) were recorded by focal animal sampling methods until their behavior changed or when they moved out of sight. A ZEISS video telescope (Victory PhotoScope 85 T*FL, 15–45×) was used to observe shorebirds and record videos of their foraging activities from a tent. The length of each observation was 5 min, and 93 focal animal videos were recorded, including 30 common greenshank, 22 spotted redshank, 20 Kentish plover, and 21 little ringed plover, respectively (File S2). For analysis, the playback speed of the focal animal videos was slowed down (0.5×) by PIPI Player.

The different species fed in bare mudflats, or in water depths that suited their morphological traits. In the video material, the water depth was calculated based on the length of the submerged part of the tarsal plantar. Common greenshank and spotted redshank birds have similar bill sizes (54.8 and 55.8 mm, respectively) and leg lengths (59.7 and 54.4 mm, respectively), as do Kentish and little ringed plovers (bill sizes 15.9 and 12.8 mm; leg length 26.4 and 23.5 mm, respectively) (Zhao, 2001).

### 2.4 Statistical analyses

Pearson's correlation was used to analyze the relationships between the mean bill size, mean leg length, foraging time ratio, foraging frequency, a foraging bout length, and the water depth of the foraging habitat (Kober, 2004). A Kolmogorov–Smirnov Z nonparametric test was used to determine differences between two data groups, and a Chi‐squared test was employed for differences between more than three data groups.

A multiple linear regression (MLR) was applied to predict the effects of the different microhabitat selection by the different species of shorebirds. The total and foraging individual number of birds were used as the response variables, and the type of microhabitat selected, as well as the foraging techniques, were the fixed predictor variables when there was a relationship with the response variables (Zuur et al., 2009). On this basis, we used “stepwise selection” to analyze the effectiveness of the predictor variable and to obtain the degree to which the predictor variable explained the response variable.

The diversity of habitats and foraging techniques was calculated using the Shannon–Wiener Index (John & James, 1988). To understand how the four shorebird species share habitat and food to avoid competition as much as possible, ecological niche overlap (*O*_*kj*_ = sum(*p*_*ij*_ * *p*_*jk*_)/sqrt(sum((*p*_*ij*_)^2^)*sum((*p*_*ik*_)^2^))) was calculated using Pianka's index (Pianka, 1974), where *O*_*kj*_ is the resource overlap between species *k* and *j*, and *p*_*ij*_ represents the proportion of resource *i* that is used by species *j*. Pianka's index ranges from 0 to 1, the use of habitat and foraging techniques between shorebirds are more similar when Pianka's index is close to 1.

Discriminant analysis was applied to describe interspecific differences induced by microhabitat use and foraging techniques. The species code (COGR: common greenshank, code 1; LIRI: little ringed plover code 2; KEPL: Kentish plover, code 3; SPRE: spotted redshank, code 4) was used as the grouping variable, all habitat types (H1–7) and foraging techniques (PE, MPE, PR, and MPR) were used as predicted variables. A significance value of *p* ≤ .05 was set in tests of equality of group means between grouping variable and predicted variables. We then obtained canonical discriminant function coefficients and used a cross‐validation technique to verify the accuracy of the discriminant analysis (Poisbleau et al., 2010).

All analyses were conducted using SPSS version 23. The significance level of all statistical tests was ≤0.05, and results were stated as the mean ± standard deviation.

## 3 RESULTS

### 3.1 Microhabitat use

**FIGURE 3:**
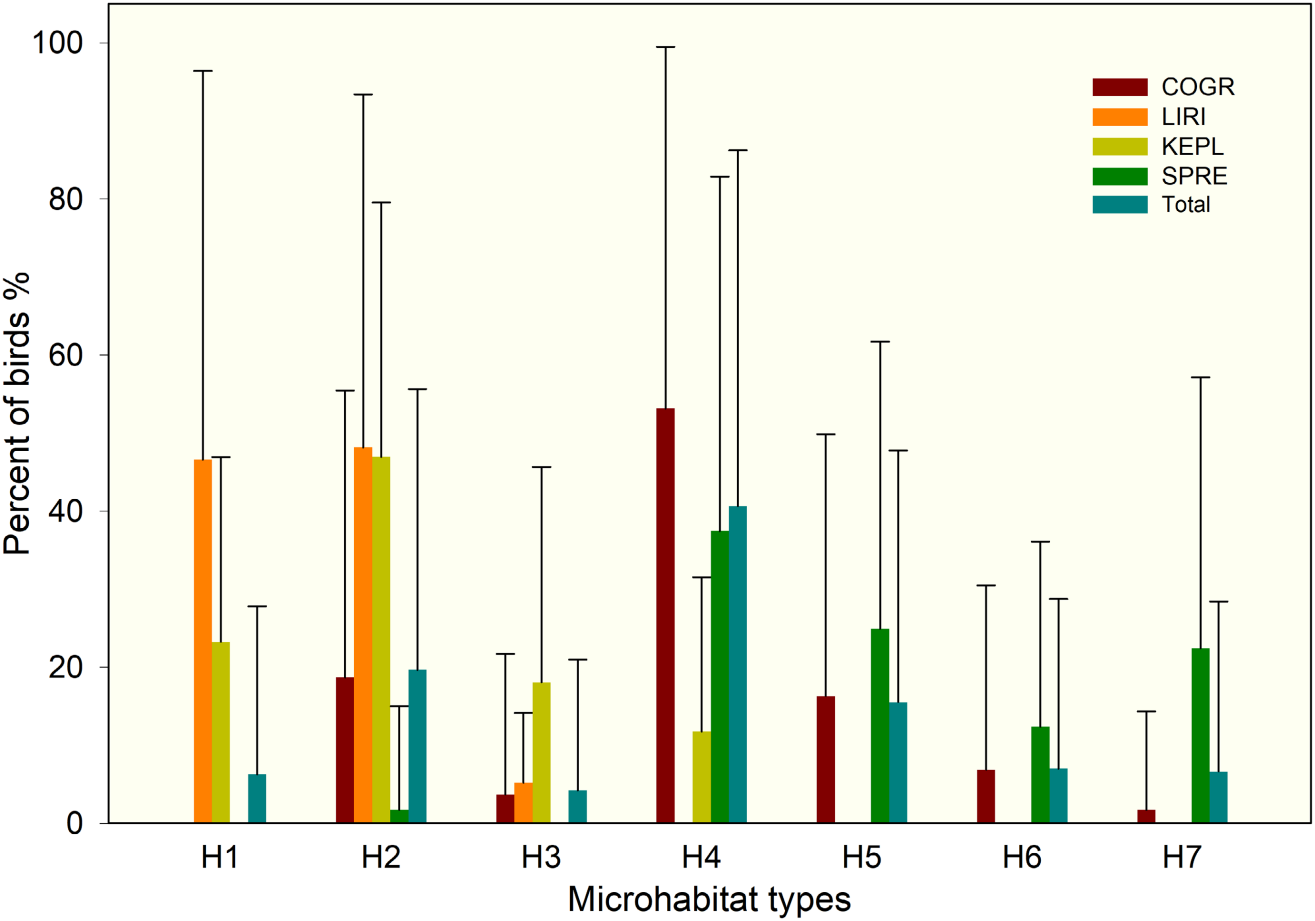
Foraging microhabitats used by shorebirds in the Shengjin Lake. H1: vegetated area; H2: dry mud; H3: soft mud; H4: shallow water; H5: intermediate water depth; H6: deep water; H7: water body. COGR, common greenshank; KEPL, Kentish plover; LIRI, little ringed plover; SPRE, spotted redshank.

Common greenshank and spotted redshank birds foraged in six and five of the seven microhabitats, respectively. Common greenshanks did not forage in the vegetated area (H1). Spotted redshanks foraged in dry mud or habitats with water depths >0.5 cm (H4–H7). Kentish and little ringed plovers did not forage in shoal areas with water depths >3 cm (H5–H7). The occurrence of mixed‐species flocks varied based on the habitat type, and five mixed flocks were found: H1: Kentish plover and little ringed plover; H2: all four species mixed; H3: three species, excluding spotted redshank; H4: three species, excluding little ringed plover; H5–H7: common greenshank and spotted redshank (Figure 3).

The habitat types used by these four species were different. The shorebird count percentage of common greenshank and spotted redshank differed in the water body (*Z* = 2.056, *p* < .001), as well as in soft mud (*Z* = 1.362, *p* = .049). Kentish plover and little ringed plover only differed in the vegetated area (*Z* = 1.44, *p* = .032) (Figure 3).

Kentish and little ringed plovers were most similar in their microhabitat preference, and hence the overlap index of microhabitats used by them was the highest. However, the overlap index of Kentish plovers and spotted redshank was 0.15, and that of Kentish plovers and common greenshank was 0.36 (Table 1). The overlap indices of the microhabitats used by the spotted redshank and common greenshank were very high; however, the overlap indices of spotted redshank and both Kentish plover as well as little ringed plover were 0.01 (Table 1). We found a negative relationship between the Pianka's index and bill length (*r* = −.940, *p* = .005) as well as Pianka's index and leg length (*r* = −.895, *p* = .016).

**TABLE 1:** Pianka's indices of overlap in microhabitat use and Shannon–Wiener diversity indices of foraging microhabitats of the spotted redshank (SPRE), common greenshank (COGR), Kentish plover (KEPL), and little ringed plover (LIRI).

	Species	COGR	SPRE	KEPL	LIRI
Pianka's index	COGR	–	0.78	0.36	0.15
	SPRE		–	0.01	0.01
	KEPL			–	0.95
Shannon–Wiener diversity index		1.17	1.32	1.28	0.88

A MLR was employed to predict the selection of foraging microhabitats by the shorebird species. All of the foraging techniques and microhabitat types (H1‐7) excluding the Deep water (H6) were used in the model. The variables Shallow water (H4), PE, Soft mud (H3), Vegetated area (H1), Water body (H7), Intermediate water depth (H5), and Dry mud (H2) predicted the total individual number, and the Shallow water (H4) was the most important (adjusted *R* ^2^ = .527) (*F* = 251.412, *p* = .000) (File S1).

### 3.2 Relationship between foraging techniques, foraging behaviors, and functional traits

**FIGURE 4:**
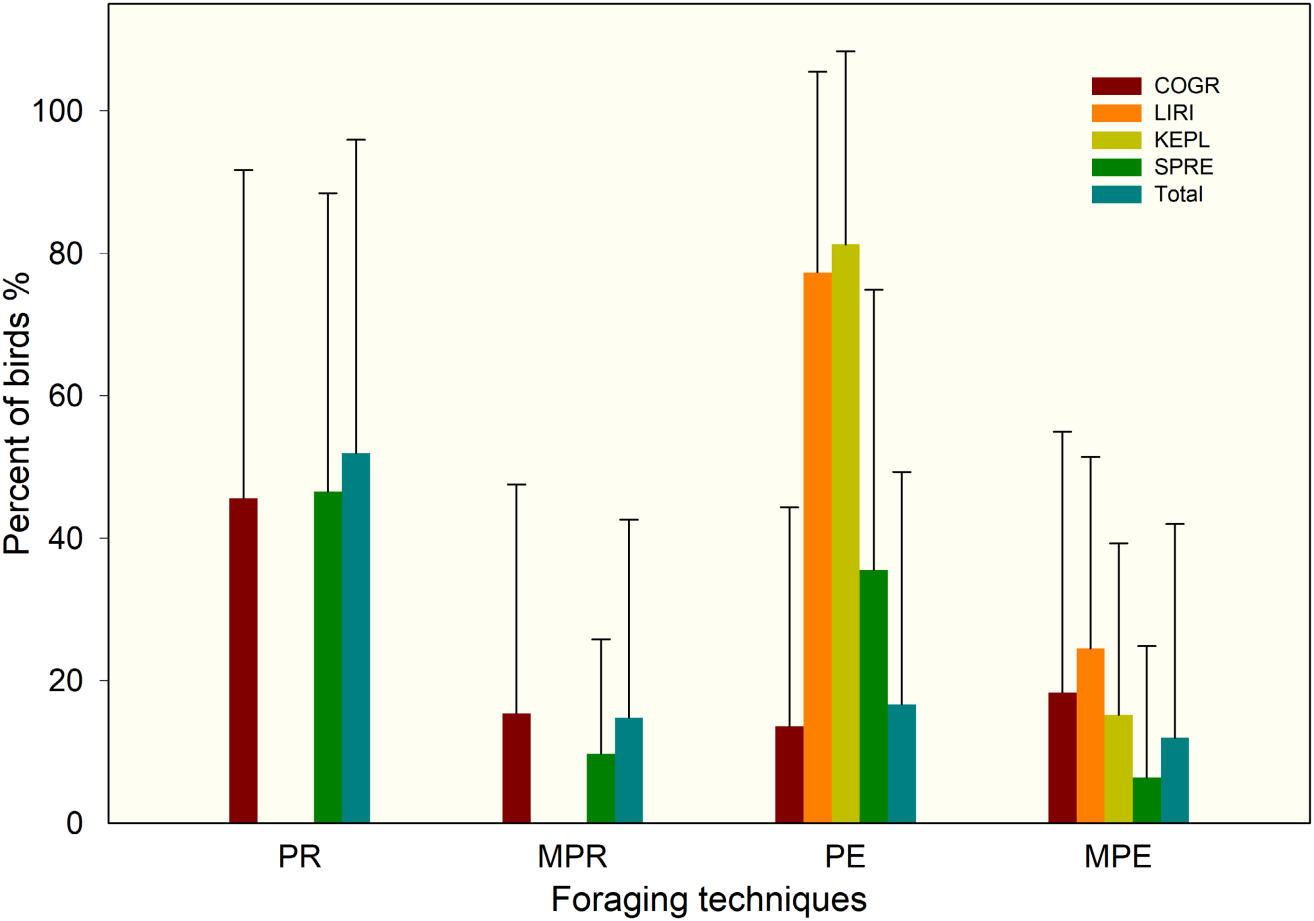
Foraging techniques used by shorebirds at Shengjin Lake. Foraging techniques: a single probe (PR); multiple probes (MPR); a single peck (PE); multiple pecks (MPE). Bird species: COGR, common greenshank; KEPL, Kentish plover; LIRI, little ringed plover; SPRE, spotted redshank.

Common greenshank and spotted redshank used four foraging techniques, showed similarities in morphological characteristics, and their overlap index was 0.89 (Table 2). All species used PE and MPE foraging techniques (Figure 4). Only the PE technique was used less frequently by common greenshank than spotted redshank (*Z* = 2.238, *p* = .000).

**TABLE 2:** Pianka's indices for the overlap in foraging techniques and Shannon–Wiener diversity indices of foraging techniques for spotted redshank (SPRE), common greenshank (COGR), Kentish plover (KEPL), and little ringed plover (LIRI).

	Species	COGR	SPRE	KEPL	LIRI
Pianka's indices	COGR	–	0.89	0.41	0.44
	SPRE		–	0.73	0.73
	KEPL			–	0.98
Shannon–Wiener diversity indices		1.28	1.14	0.41	0.58

The little ringed plover and Kentish plover used PE and MPE foraging techniques, and the overlap index of foraging techniques between them was the highest (0.98) (Table 2). Moreover, the usage rates of these two foraging techniques did not vary between species (File S1).

The results of the Kolmogorov–Smirnov *Z* test showed significant differences between the species in terms of foraging frequency (*χ* ^2^ = 51.748, *p* < .001), a foraging bout length (*χ* ^2^ = 44.566, *p* < .001), and water depth (*χ* ^2^ = 68.378, *p* < .001) but not in the foraging time ratio (*χ* ^2^ = 2.831, *p* = .418). Between the species, the foraging frequency by common greenshank (15.91 frequency/5 min) was lower than that of spotted redshank (23.88) (*Z* = 1.717, *p* = .006), whereas a foraging bout length (1.54 s vs. 0.93 s) was higher (*Z* = 1.425, *p* = .034). Foraging frequency (19.28 ± 10.17 vs. 39.87 ± 9.85 frequency/5 min) (*Z* = 4.11, *p* < .001) and a foraging bout length (1.3 ± 0.9 s vs. 0.52 ± 0.22 s) (*Z* = 3.23, *p* < .001) of the larger‐sized group of birds (common greenshank and spotted redshank) was lower than that of the smaller‐sized group (Kentish plover and little ringed plover), however, the water depth (3.7 ± 2.3 cm vs. 0.2 ± 0.2 cm) was greater (*Z* = 4.79, *p* < .001) (File S2).

The mean bill size (*r* = .993, *p* = .007), mean leg length (*r* = .996, *p* = .003), and mean foraging frequency (*r* = −.982, *p* = .000) were significantly associated with the water depth of the bird's foraging habitats. Kentish plover's a foraging bout length was positively correlated with water depth (*r* = .482, *p* = .031); these two variables were negatively correlated for little ringed plover (*r* = −.669, *p* = .001); however, the foraging frequency for both was positively correlated with water depth (*r* = .723, *p* = .000).

The mean bill size (*r* = −.953, *p* = .047) and mean leg length (*r* = −.982, *p* = .018) were significantly correlated with the mean foraging frequency for shorebirds. The foraging frequency was significantly correlated with a foraging bout length (*r* = −.963, *p* = .037). Additionally, the foraging frequency was only correlated (*r* = −.479, *p* = .028) for little ringed plover and common greenshank.

The MLR was applied to predict the foraging individual number. The foraging techniques and microhabitat type excluding Dry mud (H2) and Deep water (H6) were used in the model. PE, PR, Vegetated area (H1), and MPR variables completely predicted foraging individual number, and the PE variable was the most important (adjusted *R* ^2^ = .513) (*F* = 235.69, *p* = .00) (File S1).

### 3.3 Relationship between foraging techniques and microhabitat variables

Three typical functions were generated from discriminant analysis (Table 3). The vegetated area was the most important variable of the first function, the most important variable in the second function and the third function was water body and dry mud; however, the low level of variance indicated that they were not of great significance (Table 4).

**TABLE 3:** Summary of the canonical discriminant functions. The total of variance for three functions reached 100%, the first function is the most important of these functions.

Function	Eigenvalue	Variance (%)	Total variance (%)	Canonical correlation
1	2.6085	87.73	87.7	0.85
2	0.2138	7.191	94.9	0.419
3	0.1510	5.079	100.0	0.362

**TABLE 4:** Canonical loadings of the discriminant functions affecting shorebird groups, which excluded Multiple probes (MPR) foraging technique and the Shallow water (H4) microhabitat type; Vegetated area (H1) was the important variable.

Variable type	Function 1	Function 2	Function 3
Foraging technique			
PR	−0.062	0.091	−0.007
PE	0.219	−0.156	−0.325
MPE	−0.075	−0.080	0.359
Microhabitat			
Vegetated area (H1)	0.561	0.291	0.029
Soft mud (H3)	0.199	0.185	0.260
Dry mud (H2)	0.329	−0.358	−0.512
Intermediate water depth (H5)	−0.139	0.214	0.143
Deep water (H6)	−0.170	0.346	0.041
Water body (H7)	−0.251	0.512	0.026
(Constant)	−0.544	−0.529	0.065

**FIGURE 5:**
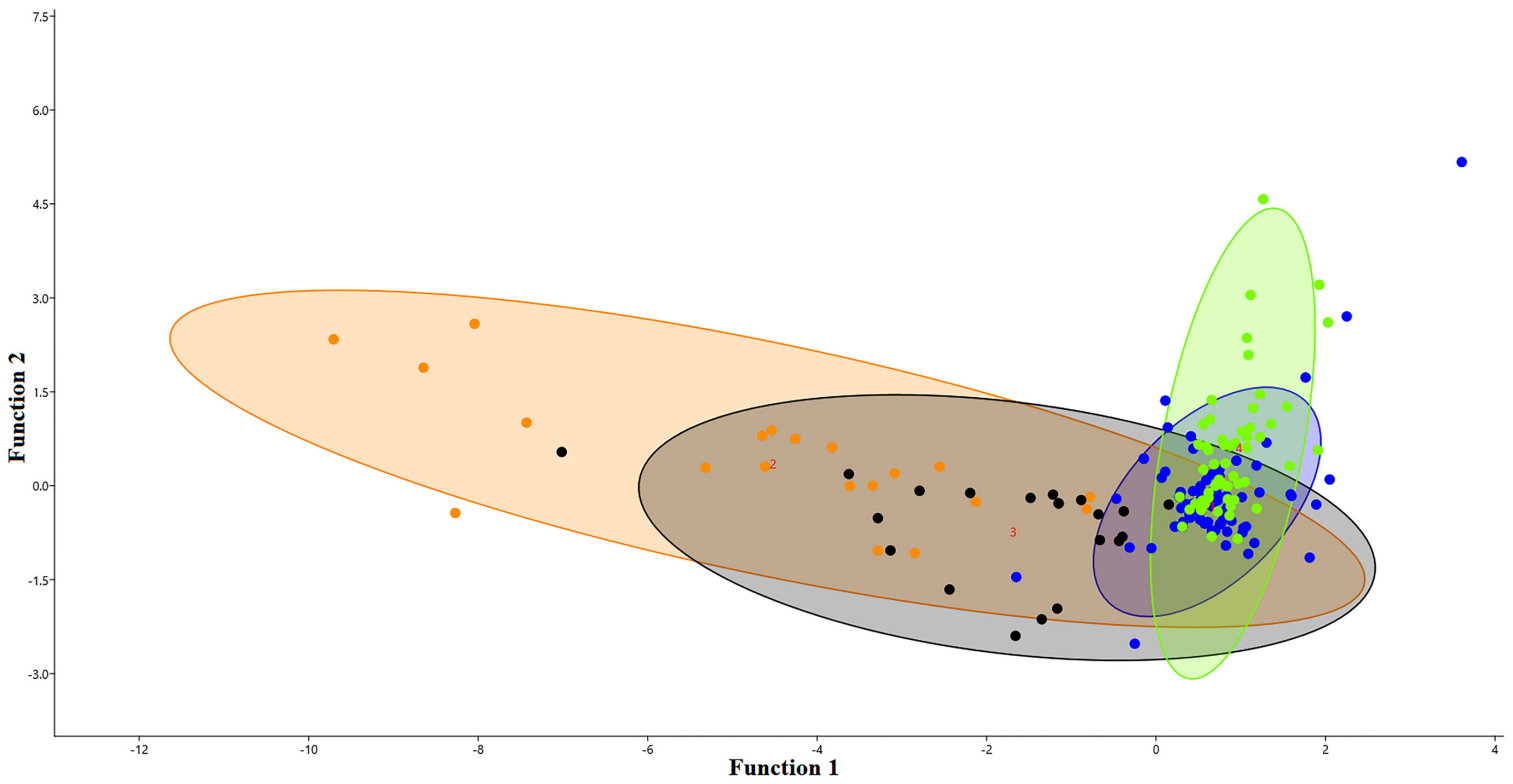
Results of the discriminant analysis. The colors blue, green, black, and brown represent common greenshank (COGR, code 1), spotted redshank (SPRE, code 4), Kentish plover (KEPL, code 3), and little ringed plover (LIRI, code 2), respectively. The solid curve lines represent the statistical boundary between different groups.

The large‐sized bird group (common greenshank and spotted redshank) had higher scores than the small‐sized group (little ringed plover and Kentish plover), suggesting that the large birds chose different microhabitats and foraging techniques (Figure 5). The original classification of the whole sample showed that 76.1% of the samples were classified correctly, and cross‐validation showed that 73.9% were classified correctly, which is consistent with the obtained original accuracy.

## 4 DISCUSSION

In this study, it was found that four shorebird species at Shengjin Lake overlapped in their selection of foraging microhabitat and in their use of foraging techniques, to a certain extent. This study demonstrated niche separation of shorebirds at a wintering site.

The mechanism for mixing among these species of shorebirds was reflected in their spatial coexistence, which was largely related to their characteristics, as has been shown in previous studies (Eckhardt, 1979; Engelhardt et al., 2014). The different characteristics determine the functional differences in foraging organs, which leads to differences in foraging techniques, and hence, different foraging techniques are applicable to different substrate types in the habitat. Therefore, the different characteristics indirectly result in divergent spatial distribution patterns, leading to stable spatial mixing of the different species.

In the findings of this study, the use of foraging techniques resulted in a certain degree of separation among all four species. There was low separation between common greenshank and spotted redshank, which had similar morphologies. Little ringed plover and Kentish plover did not use probing or multiple probing foraging techniques, therefore showing low overlap with common greenshank in the selection of foraging microhabitats, which was consistent with the findings of studies on the ecological and morphological features of these species (Bocher et al., 2014). However, common greenshank and spotted redshank showed great overlap in their selection of foraging microhabitats, had similar diversity indices, and also used several foraging techniques. They may have adopted their foraging strategies opportunistically and switched between various foraging techniques. Any subtle differences in the morphological characteristics, such as the bill and leg lengths, may lead to different foraging techniques in these shorebirds, as has been reported before (Olsen, 2017).

The increase in bill sizes, for example, allows birds to forage in deep mud or water environments. Short‐billed birds tend to insert most or all of their bills into the mud or water while foraging. Long bills allow birds to probe deeper in the mud when they feed. This study showed that common greenshank and spotted redshank tended to forage in deep mud and in areas near shallow water. Kentish plover and little ringed plover often used mudflats that were close to the vegetated area, which were shallower and drier than the areas closest to shallow water. Similar findings indicate that shorebirds with short legs are restricted to mudflats or shallow waters along the edge of a wetland (Norazlimi & Ramli, 2015). The present study found that leg length was also positively correlated with water depth in the shorebirds selected. If characteristics associated with foraging of these species are similar, then the diversity of foraging techniques may be an important mechanism for determining foraging‐based niche separation among different species. The species observed during this study are small, short‐legged, and short‐billed; however, they have relatively large eyes, and their bills are hard, all these characteristics may affect their foraging technique and habitat selection. Due to the available focal species pools at our study site, larger plovers and smaller sandpipers were not included. Therefore, colinearity among many morphological traits may limit the scope of this study and the applicability of our results. In future studies, more morphologically similar and disparate species should be included in the species pool.

In terms of the time dimension, the mechanism of mixing is closely related to the conditions of the substrate, patch size, and water depth. First, exposure of the mudflat zone follows a certain pattern in winter, which depends on natural hydrological processes or artificial water level regulation. Size, habitat, food type, and time are the most important resource axes of species separation, and their importance is in descending order (Polla et al., 2018; Schoener, 1974). Several studies on ecological niche separation have shown that the separation in food types among many migratory species plays a critical role and is related to size (Jia et al., 2019). Single predators or communities leave when their food becomes depleted beyond a certain point, resulting in differences in residence times and giving rise to mixed populations and time separation in a habitat. In this study, it was unfortunately not possible to thoroughly investigate the effects of food type on the foraging niches of shorebird species as it was impossible to determine the specific types of food that the birds were preying on. The observation time was also limited to 6 months. Food type and its effect on residence times could be further investigated in future studies.

## 5 CONCLUSIONS

Our study contributes to the findings that the coexistence of shorebirds is regulated by the selection of different habitats, which is influenced by their morphological characteristics and specific conditions in the wintering habitats, such as hydrological rhythms. Moreover, the morphology of shorebirds plays an important role in determining foraging techniques and foraging behaviors. Therefore, the diversity of microhabitat types should be considered in habitat restoration and management strategies to improve the survival of wintering birds in the area. The results of this study could be applied to the management of Shengjin Lake and the shorebirds that winter there. Because of the differences in body size, bills and legs, shorebirds such as the common greenshank, spotted redshank, little ringed plover, and Kentish plover have various spatial mixing patterns in waters with different depths. Moreover, the water flow is controlled by the artificial gate of Shengjin Lake, which affects the water depth. Therefore, to provide better habitat patches for the shorebirds at Shengjin Lake, the managers in charge of the sluice gates should adjust the water level at specific times according to the distribution and location of the shorebirds to satisfy their habitat and foraging needs.

## AUTHOR CONTRIBUTIONS

**Chao Yu:** Data curation (equal); investigation (equal); methodology (equal); writing – original draft (equal). **Ruilin Zhang:** Data curation (equal); investigation (equal); methodology (equal); writing – original draft (equal). **Lizhi Zhou:** Conceptualization (equal); funding acquisition (equal); supervision (equal); writing – review and editing (equal). **Lei Cheng:** Software (equal). **Yiwei Bao:** Software (equal). **Yunwei Song:** Investigation (equal).

## Supporting information


File S1.

File S2.

## Data Availability

Data are provided as Files S1 and S2.
